# A Randomized Clinical Trial of Inhaled Nitric Oxide Treatment in Premature Infants Reveals the Effect of Maternal Racial Identity on Efficacy

**DOI:** 10.3390/jcm12247567

**Published:** 2023-12-08

**Authors:** Jeremy D. Marks, Michael D. Schreiber

**Affiliations:** Departments of Pediatrics and Neurology, University of Chicago, Chicago, IL 60637, USA; jmarks@uchicago.edu

**Keywords:** bronchopulmonary dysplasia, outcomes, prematurity, respiratory distress syndrome

## Abstract

Respiratory distress syndrome increases the risk of death and bronchopulmonary dysplasia (BPD) in premature infants. Inhaled nitric oxide (iNO) may reduce these risks. Recent meta-analyses have suggested that iNO is effective only at doses higher than 5 ppm and in infants born to Black mothers. In a randomized, double-blinded, controlled trial, infants born before 32 0/7 weeks gestation, weighing <1500 g, and requiring respiratory support were assigned to receive iNO for either seven days (short iNO), or until 33 0/7 weeks PMA (long iNO). The primary outcome was death or BPD. A total of 273 patients were enrolled, of whom 83 receiving long iNO (61.5%) experienced the primary outcome, compared with 65 (47.1%) receiving short iNO (relative risk (RR) 1.37; 95% confidence interval (CI), 1.06–1.79; *p* = 0.017). This increase was due solely to increased BPD in infants weighing 750–999 g (RR 1.33, 95% CI 1.07–1.66, *p* = 0.009). However, there was no difference in the numbers of infants requiring supplemental oxygen at 40 weeks PMA. Among infants < 750 g, long-iNO-treated infants had a lower cumulative probability of death (χ^2^ 5.12, *p* = 0.02). Long iNO increased the primary outcome in non-Black infants (RR 1.93, 95% CI 1.20–3.24) but not in Black infants. Understanding how maternal racial identity determines responses of premature infants to iNO may help narrow the gap in health outcomes between Black and non-Black infants.

## 1. Introduction

The severity of lung disease in preterm infants increases the birth-weight-dependent risk of death, bronchopulmonary dysplasia (BPD), and adverse long-term neurodevelopmental outcomes [1,2]. Inhaled nitric oxide (iNO), first used as a pulmonary vasodilator in full-term infants with increased pulmonary vascular resistance, has multiple beneficial effects on the developing lung [3], and iNO treatment of preterm infants requiring respiratory support may reduce death and BPD through these effects.

Results of large, placebo-controlled, double-blind, single- and multi-center trials of iNO treatment to reduce death and BPD in premature infants have been mixed. No respiratory or survival effects of iNO treatment have been found in studies employing starting iNO concentrations of <10 ppm or employing treatment durations of <7 days [4,5,6]. In contrast, two randomized, double-blind, placebo-controlled trials employing starting iNO concentrations greater than 5 ppm, with iNO treatment lasting at least seven days, have found iNO treatment to reduce death or BPD [7,8], while one did not [9]. As important to the efficacy of iNO treatment in decreasing BPD and death in premature infants as starting iNO concentration and treatment duration may be the racial identity of the patient receiving iNO: Thus, a meta-analysis employing individual patient data from the complete cohorts of the three studies above [7,8,9] recently demonstrated that iNO treatment of infants, identified by their mothers as Black, reduced the composite outcome of death or BPD, as well as survival with BPD, compared with treatment of Black infants with placebo [10]. In contrast, infants identified as white exhibited almost identical incidences of the composite outcome of death or BPD or survival without BPD, whether they received iNO or placebo. The social nature of racial identity suggests that the multiple socioeconomic, environmental, and psychosocial stressors that distinguish populations identified as Black from other populations in the US may influence an infant’s respiratory and other responses to prematurity.

In a previous, randomized, double-blind, placebo-controlled trial, we found that seven days of iNO treatment, beginning at 10 ppM on the first day of postnatal life, decreased the prespecified outcomes of death or BPD, as well as BPD alone, in preterm infants born <34 weeks gestation [8]. In the present randomized, double-blind, placebo-controlled study, we hypothesized that prolonged the treatment of premature infants with iNO of 10 ppm would decrease the primary outcome of BPD or death compared to treatment for seven days. To harmonize the duration of the longer iNO therapy with the degree of prematurity, infants in the long iNO arm of the study were treated from birth until 32 6/7 weeks post-menstrual age (PMA). Because the recent meta-analysis cited above [10] suggested that only infants of Black mothers responded to iNO with beneficial effects, we also hypothesized that the long iNO treatment would differentially benefit Black versus non-Black infants.

## 2. Methods

This was a randomized, double-blind, controlled trial involving premature infants cared for at a single, University-affiliated, tertiary care neonatal intensive care unit in the United States that admitted approximately 1000 infants per year, of whom approximately 200 infants per year weighed <1500 g at birth. The University of Chicago Hospitals’ Neonatal Intensive Care Nursery, at which the study was performed, cares for a majority (70%) Black population born to a high-risk population. Overall, over 85% of the mothers of the newborns cared for in this center are without private health insurance, and may receive state health insurance for low-income families. This study was approved by the Institutional Review Board of the University of Chicago. Written informed consent for participation of eligible infants was obtained from the mother. This trial was registered at ClinicalTrials.gov, number NCT00515281.

### 2.1. Outcomes

The primary outcome was the combined incidence of death before discharge from hospital or BPD. BPD was defined as requiring respiratory support at 36 weeks post-menstrual age (PMA), either as positive pressure or supplemental oxygen to maintain oxygen saturations greater than 90%. Predefined secondary outcomes were the incidences of (1) death; (2) BPD; (3) supplemental oxygen use at 40 weeks PMA; (4) death or BPD in infants with birthweight less than 750 g; (5) death or BPD in infants with birthweight 750–999 g; and (6) death or BPD in Black infants. Pre-specified secondary outcomes included the combined outcome of severe intraventricular hemorrhage (IVH, Grades 3 or 4) and periventricular leukomalacia (PVL), pulmonary hemorrhage, pneumothorax, pulmonary interstitial emphysema (PIE), symptomatic patent ductus arteriosus (PDA), and necrotizing enterocolitis (NEC) of Bell’s stage 2 severity or greater.

### 2.2. Criteria for Eligibility

All premature infants cared for between 2008 and 2014 at the University of Chicago Hospitals’ Intensive Care Nursery, born at less than 32 0/7 weeks gestation and without major congenital malformations, who had a birth weight < 1500 g, and who required respiratory support were screened for eligibility on the day of admission. Because iNO treatment of premature infants with severe hypoxic respiratory failure has been associated with worse outcomes [11], we excluded infants with an oxygenation index of greater than 20, defined as the fraction of inspired oxygen (×100) multiplied by the mean airway pressure, divided by the partial pressure of arterial oxygen. 

### 2.3. Study Design and Randomization

Infants were randomly assigned on the day of admission in a 1:1 ratio to one of the two following groups: (1) receiving iNO for seven days (short iNO); and (2) receiving iNO until 33 0/7 weeks PMA (long iNO). Infants were randomized within the following birthweight strata: <750 g, 750–999 g, 1000–1249 g, and 1250–1499 g. Randomization was performed according to a permuted block design. The random sequence was generated online, and opaque sealed envelopes labeled with the birth weight stratum and a numeric code were prepared in block sizes of 8. The respiratory therapist opened the next numbered envelope in the appropriate birth weight stratum to record the treatment arm to which the infant had been randomized and provide the infant with that treatment.

### 2.4. Study Protocol

Infants supported with mechanical ventilation through an endotracheal tube were provided iNO at 10 ppm for the first 24 h. After 24 h, the iNO concentration was decreased to 5 ppm. Infants supported with nasal continuous positive airway pressure (CPAP) or nasal cannula oxygen were provided an iNO concentration at twice the concentration delivered via invasive ventilation [12]. Study gas was delivered until 33 0/7 weeks PMA using an INOvent (Mallinckrodt Pharmaceuticals, St. Louis, MO, USA) that was shielded so that the arm of the study to which the infant had been randomized and the concentration of the study gas provided was known only to the respiratory therapist and the study safety monitor. In infants receiving short iNO, the iNO concentration was decreased to 0 ppm after seven days of treatment, and this placebo gas was provided through the shielded iNOvent until 33 0/7 weeks PMA. Infants in the long iNO arm continued to receive iNO until 33 0/7 weeks PMA. The study gas was delivered to each infant through the shielded iNOvent using the clinically indicated respiratory support device. Infants who no longer required respiratory support before 33 weeks PMA were provided with 21% oxygen through the shielded iNOvent (with or without iNO) via nasal cannula at 0.5 L/min. All clinical decisions were made by clinicians who, like the investigators, were unaware of any infant’s treatment assignment. 

The outcome of BPD was assigned to infants who, at 36 weeks PMA, were receiving positive pressure respiratory support, or at least 30% supplemental oxygen in the absence of positive pressure. Infants receiving <30% supplemental oxygen without positive pressure were subjected to a room air challenge. The outcome of BPD was assigned to these infants if oxygen saturations could not be maintained above 90% in room air for 30 min [13]. Racial identity was obtained by maternal report. 

### 2.5. Safety Monitoring

The safety and data monitoring committee, which was unaware of treatment assignment, analyzed the data at study midpoint and approved its continuation.

### 2.6. Statistical Analyses 

We estimated that a sample of 338 patients would provide 80 percent power to detect an absolute change of 15 percent in the 50% incidence of the primary outcome we reported in our previous study [8], with a two-sided type I error of 0.05. Study data were collected and managed using REDCap electronic data capture tools hosted at the University of Chicago. We conducted the analysis according to intention-to-treat. 

For continuous variables, differences between groups were tested with unpaired *t*-tests [14] or by the Wilcoxon rank-sum test [15] depending on the data distributions. Differences in categorical variables between groups were tested with the chi-square [16] or Fisher’s Exact test [16], depending on the number of observations in each cell. Outcomes were also analyzed using a generalized linear model with logarithmic link. Because whether infants died following hospital discharge was unknown, survival differences between groups were assessed using a model in which discharge from the hospital was treated as a competing risk [17,18]. Cumulative incidence function plots of death in hospital and discharge from hospital were derived using the non-parametric Aalen–Johansen estimator [17,19,20]. Differences between cumulative incidence functions were tested using a K-sample test statistic against a chi-square distribution [18]. All *p* values are two-sided. 

## 3. Results

**Figure 1:**
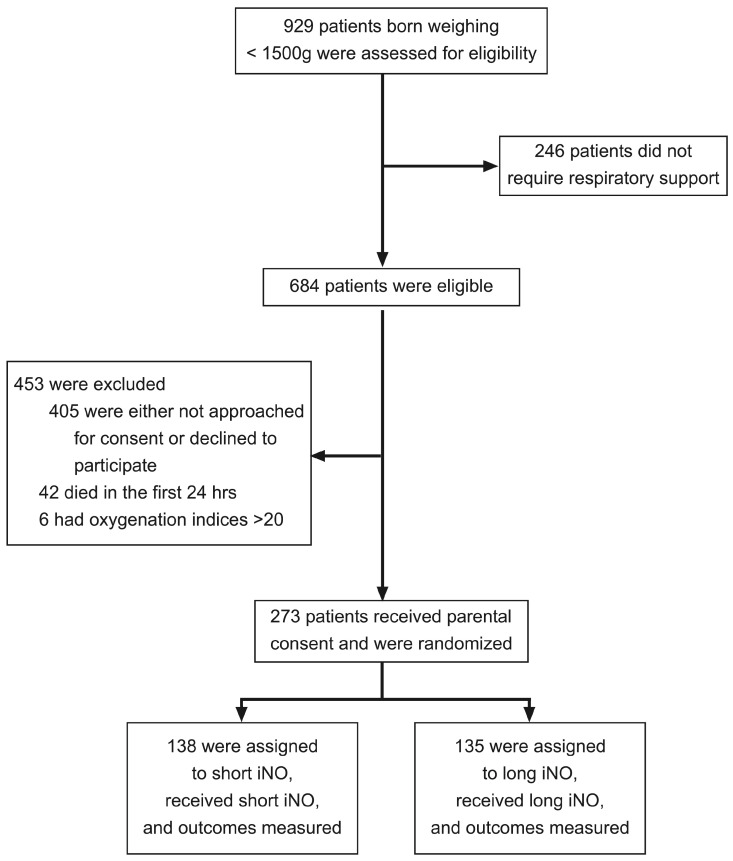
Enrollment, randomization, and outcome measurement of the patients.

A total of 273 patients were enrolled (Figure 1). The pace of patient enrollment forced recruitment to stop after these patients (81% of the desired 338 patients) had been enrolled. 

### 3.1. Patient Characteristics

Subject demographics, including birth weight, racial identity distributions, and other clinical characteristics did not differ significantly between the short iNO group and the long iNO groups (Table 1). The birthweight distribution of the enrolled cohort was as follows: 82 infants (30.0%) weighed less than 750 g, 87 (31.9%) weighed between 751 and 999 g, 67 (24.5%) weighed between 1000 and 1249 g, and 37 (13.6%) weighed between 1250 and 1499 g. Over 80% of infants in both groups were born to mothers who were known to have received antenatal steroids. Neither the incidences of chorioamnionitis in the mother nor the incidences of postnatal infection in the infants differed between the groups. The racial identity of the infants, as determined by maternal report, was as follows: 190 infants (69.6%) were identified as Black, 38 (13.9%) were identified as white, and 45 (16.5%) were identified as neither. Analyses of associations between outcomes and racial identity compared infants identified by their mothers as Black with the group consisting of infants identified by their mothers as white, plus those who were identified as neither, i.e., non-Black. There were 83 infants (30.4%) who were non-Black.

### 3.2. Primary Outcome

A total of 65 of 138 infants (47.1 percent) in the short iNO group and 83 of 135 infants (61.5 percent) in the long iNO group died or developed BPD (relative risk with long iNO, 1.37; 95% confidence interval (CI), 1.06–1.79; *p* = 0.017). The incidence of death did not differ between the groups (Table 2). Thus, the difference in the primary outcome between the groups was entirely due to the increased incidence of BPD in the long iNO group: 51 of 138 infants (37.0 percent) in the short iNO group and 71 of 135 infants (52.6%) developed BPD (relative risk with long iNO, 1.33; 95% CI, 1.07–1.66). 

Many infants meeting the criteria for BPD at 36 weeks gestation no longer require supplementary oxygen at 40 weeks gestation. Babies in this study receiving supplemental oxygen at 36 weeks PMA were not discharged until after 40 weeks PMA or later, allowing for the determination of the incidence of the need for supplemental oxygen at 40 weeks PMA. There was no difference in the post-menstrual age at discharge between the treatment groups (short iNO: median 42.57 weeks, IQR: 41.07–48.15; long iNO: median 41.86 weeks, IQR: 39.12–46.0; *p* = 0.21). Importantly, there was no difference between the groups in the number of infants still receiving supplemental oxygen at 40 weeks PMA (short iNO: 30 of 134 infants (22.4%); long iNO: 28 of 126 infants (22.2%); relative risk with long iNO, 0.97; 95% CI 0.87–1.14, *p* = 0.97, Table 2). Accordingly, although long iNO increased the risk of BPD at 36 weeks PMA compared with short iNO treatment, the increased risk of being on supplemental oxygen in long-iNO-treated infants had disappeared by 40 weeks gestation. 

### 3.3. Birth Weight-Specific Effects on the Primary Outcome

Because the incidences of death and bronchopulmonary dysplasia decrease as birth weight and gestational age increase, we compared the incidences of the primary outcome between the treatment groups across the different birth weight groups. We found no differences in infants weighing in the range of 1000–1249 g or weighing more than 1249 g at birth (Table 3). Importantly, there was also no difference in the incidences of the primary outcome between the short iNO and long iNO groups in infants weighing <750 g at birth: 36 of 41 infants (87.8 percent) in the short iNO group died or had BPD compared to 37 of 41 infants (90.2 percent) in the long iNO group (relative risk, 1.0; 95 percent confidence interval, 0.9 to 1.2; *p* = 0.72, Table 3). In contrast, for infants born in the range of 750–999 g, the primary outcome was unexpectedly increased in the long iNO group (Table 3). In the short iNO group, 20 of 43 infants (46.5 percent) died or developed BPD compared to 36 of 44 infants (81.8 percent) in the long iNO group (relative risk, 2.94; 95 percent confidence interval, 1.54–5.90; *p* < 0.001). In the logistic regression analysis of the primary outcome by weight group and treatment assignment, there was no significant interaction between birth weight group and treatment (*p* = 0.47).

### 3.4. Components of the Primary Outcome in Infants Weighing <1000 g at Birth

In infants weighing 750–999 g at birth, in whom the incidence of the primary outcome was increased in the long iNO group, there was no difference in the incidences of death between the two treatment groups. Accordingly, the increase in the primary outcome in this birth weight group treated with long iNO was entirely due to the increased incidence of BPD. Thus, in the long iNO group, 30 of 44 infants (68.2%) had BPD compared with 17 of 43 infants (39.5%) in the short iNO group (relative risk 1.90, 95% CI 1.18–3.17, *p* = 0.007, Table 4). Notably, there was no difference in the number of infants on supplemental oxygen at 40 weeks post-menstrual age (short iNO: 9 of 42 infants (21.4%); long iNO: 13 of 38 infants (21.3%; relative risk 1.19, 95% CI 0.91–1.62, *p* = 0.20). 

Unlike the infants weighing 750–999 g at birth, long iNO treatment of infants born <750 g increased neither the incidence of the primary outcome nor the incidence of BPD alone compared to those treated with short iNO (short iNO: 36 (87.8%) of 41 infants; long iNO: 37 (90.2%) of 41 infants, *p* = 0.72; Table 5). Thus, our observation of an increased incidence of BPD in the entire cohort treated with long iNO was solely due to its increased incidence in the group of larger infants weighing 750–999 g at birth. Notably, in infants weighing <750 g at birth, long iNO treatment halved the incidence of death observed in the short iNO group (short iNO 14 (34.1%) of 41 infants; long iNO: 7 (17.7%) of 41 infants; relative risk for long iNO: 0.80 (0.59–1.03; *p* = 0.08). The median age in days at which infants in each group died was not different (short iNO: 22.5 days; 9–137 days, IQR; long iNO: 27 days; 15–297 days, IQR: *p* = 0.65). 

**Figure 2:**
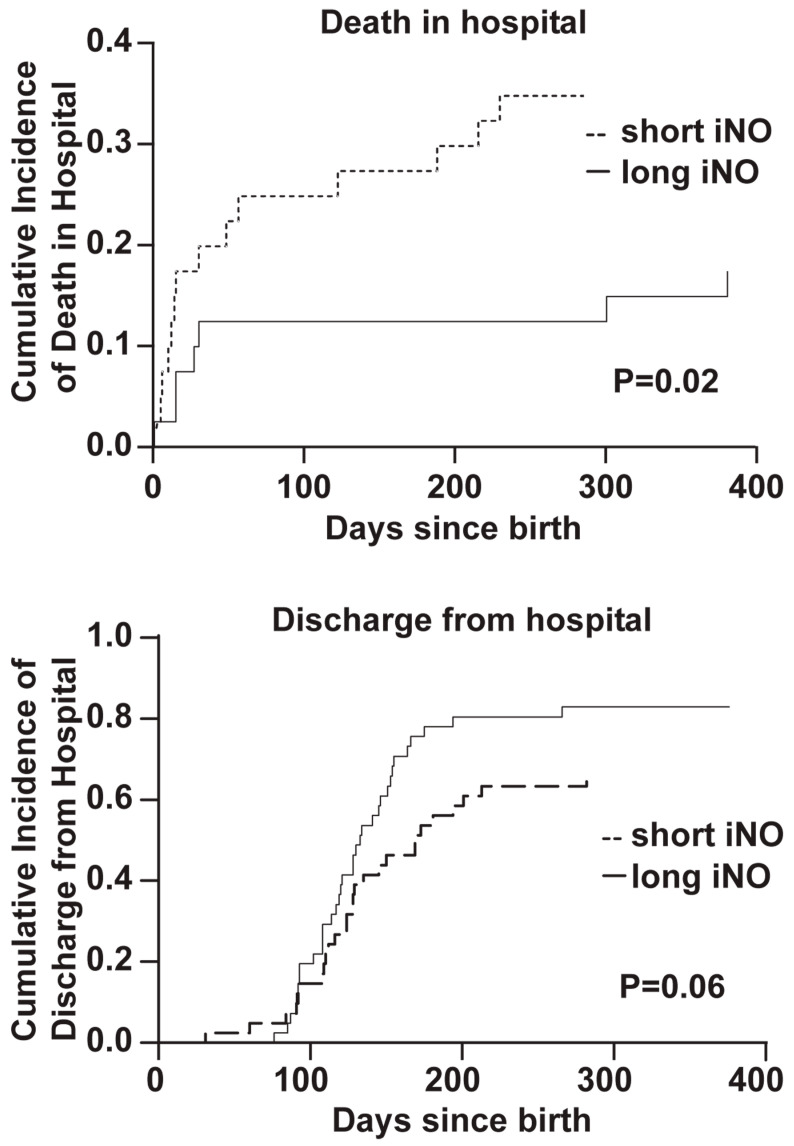
The cumulative incidences of death in hospital and discharge from hospital, treated as competing risks in infants born <750 g treated with short and long iNO.

We next compared in-hospital death between the groups, treating discharge from hospital as a competing risk [17,20]. Among these infants born <750 g, the cumulative probability of death in hospital was significantly lower in the long iNO group compared to the short iNO group (modified chi square 5.12, df = 1, *p* = 0.02, Figure 2, top). Similarly, the cumulative probability of discharge from hospital was higher in the long iNO group compared with the short iNO group (modified chi square 3.43, df = 1, *p* = 0.06, Figure 2, bottom). Thus, long iNO treatment appears to decrease death in infants born <750 g. There were no differences between the groups in either the birthweights of these surviving infants or their lengths of stay until discharge.

### 3.5. Association of the Primary Outcome and Its Components with Racial Identity

Before determining whether the length of iNO treatment differentially affected the incidences of the primary outcome and its components as a function of maternal racial identify, we asked whether maternal racial identity was associated with differences in these outcomes when the long and short iNO groups were combined. We found no such associations (Table 6). Accordingly, differences in outcomes between short- and long-iNO-treated groups categorized by racial identity reported here below are not confounded by outcome differences associated with racial identity that are unrelated to the length of iNO treatment.

In the cohort of infants whose mothers identified as non-Black, those treated with long iNO had an increased incidence of the primary outcome compared with the short iNO group (long iNO: 30 (68.2%) of 44 infants; short iNO: 15 (38.5%) of 39 infants; relative risk for long iNO: 1.93, 95% CI 1.20–3.24, *p* = 0.007, Table 7, top). There was no difference in the incidences of death between the treatment groups Accordingly, the difference in primary outcome in non-Black infants in the long iNO group was entirely due to the increased incidence of BPD (long iNO: 26 (59.1%) of 44 infants; short iNO: 12 (30.8%) of 39 infants, relative risk 1.69, 95% CI 1.14–2.61, *p* = 0.01, Table 7, top). There was similarly no difference between the treatment groups in infants of non-Black mothers in the incidence of treatment with supplemental oxygen in hospitalized infants at 40 weeks PMA.

In marked contrast to non-Black infants, long iNO in Black infants did not increase the incidences of the primary outcome or its components compared with short iNO (Table 7, bottom). Accordingly, the increased risk of BPD in the entire cohort treated with long iNO was due to BPD increases in non-Black infants. Multiple logistic regression analysis of the primary outcome using racial identity and iNO treatment length as independent variables showed a trend towards an interaction between long iNO treatment and Black racial identity (odds ratio: 0.40 95% CI 0.13–1.15, *p* = 0.09).

### 3.6. Birth Weight-Specific Effects within Racial Identity Groups

We next asked whether infants < 750 g born to Black mothers were preferentially protected from death by long iNO treatment. In infants < 750 g born to non-Black mothers, the incidences of the primary outcome, death, or BPD did not differ between the long and short iNO treatment groups (Table 8). In contrast, in infants < 750 g born to Black mothers, the incidence of death in the long iNO group was less than half of the incidence in short iNO group (short iNO: 12 (36.3%) of 33; long iNO: 5 (16.7%) of 30; relative risk of death: 0.76, 95% CI (0.55–1.03), *p* = 0.08, Table 8). In these infants, there were also no differences in the incidences of the primary outcome or BPD between short and long iNO treatment groups. These observations suggest that the protective effect of the long iNO treatment in infants < 750 g observed in the entire cohort may be restricted to infants born to Black mothers. 

### 3.7. Secondary Outcomes

The incidences of severe intraventricular hemorrhage and periventricular leukomalacia in the entire cohort did not differ significantly between the long and short iNO groups (Table 9), which is consistent with our previous work [7]. The incidences of pneumothorax, pulmonary interstitial emphysema, pulmonary hemorrhage, symptomatic patent ductus arteriosus and necrotizing enterocolitis also did not differ significantly between the groups (Table 9).

## 4. Discussion

In this study of inhaled nitric oxide treatment of very low birth weight (<1250 g) preterm infants, we tested two primary hypotheses: First, that iNO therapy, provided continuously from admission until 33 0/7 weeks post-menstrual age (long iNO), reduces the primary outcome of death or BPD compared with iNO treatment for seven days (short iNO); and second, that any benefit from long iNO treatment is restricted to infants whose mothers identify as Black. Unexpectedly, we found that long iNO treatment increased the primary outcome compared with short iNO treatment. This increase was entirely due to increased BPD alone in non-Black infants weighing 750–999 g at birth. However, by the time these infants reached 40 weeks PMA, there was no difference in the use of supplemental oxygen between long and short iNO groups. 

Although long iNO treatment did not decrease the incidence of the primary outcome or its components in the entire cohort, we found that long iNO decreased mortality in infants weighing <750 g at birth. Importantly, when we analyzed Black and non-Black infants in this birth weight subgroup separately, we found that this effect of long iNO treatment was seen only in Black infants. Because this study was not powered to detect differences between these subgroups, this finding should be viewed as hypothesis generating.

The present finding of a higher incidence of BPD in the long iNO group compared to the short iNO group was unanticipated. This increase was driven entirely by the BPD increase in infants weighing 750–999 g. In our previous trial, placebo-treated infants weighing 750–999 g had a 62.1% incidence of BPD or death, higher than both the 50.0% incidence in that study’s iNO-treated group and the 46.5% incidence we report in this study’s short iNO arm. The present finding raises questions about the effect of prolonged iNO exposure on the developing lung. Of note, the multicenter NO-CLD trial treated preterm infants with iNO or placebo for 24 days, beginning between day 7 and 21, and demonstrated that iNO increased survival without BPD compared to placebo [7]. Accordingly, it is difficult to assign the cause of the increased BPD incidence to treatment duration alone. 

In the present study, we compared long iNO with short iNO because in a randomized, double-blind, placebo-controlled trial [8], we had found that, compared to the placebo, seven days of iNO treatment decreased the combined outcome of death or BPD, survival without BPD, the combined incidence of severe intraventricular hemorrhage and periventricular leukomalacia, and, in a two-year follow-up study, decreased abnormal neurodevelopmental outcomes [21]. Accordingly, for the purposes of this research study, we felt it unethical to withhold therapy we had previously shown to be beneficial to this population. Notably, the incidences of the primary outcome and its components reported here in the short iNO group closely match the incidences we reported in our previous study for infants treated with the identical iNO regimen [8]. In the present study, the 47% incidence of BPD or death in the short iNO group is almost identical to the 48.6% we previously reported for the seven-day iNO group, which was significantly lower than the 63.7% in the control group. Similarly, the 13% incidence of death we report here in the short iNO group is nearly identical to the 15.2% incidence for the infants in the iNO arm of the previous study and not different from the 22.5% incidence in the placebo arm. Accordingly, in providing short iNO rather than placebo to our control group of infants, we were able to provide the same benefits that we had previously reported for what was then the experimental group.

We provided iNO to non-intubated infants at double the concentration used for intubated infants [12], an approach that has been shown to deliver iNO concentrations to the posterior pharynx of non-intubated patients that are not different from the concentration delivered via endotracheal tube at half the set concentration [12]. Accordingly, it is likely that the concentrations of iNO reaching the lungs of non-intubated patients in our study were similar to the concentrations delivered to intubated patients. More recently, a very large retrospective study of newborns treated at a single center (10,895 infants screened) over a period of 9 years found that, over the 24 h following the initiation of non-invasively delivered iNO, fractional oxygen requirements significantly decreased, and oxyhemoglobin saturations significantly increased [22]. These data indicate that the non-invasive provision of iNO improves oxygenation, consistent with its delivery to the pulmonary vasculature and, when provided at twice the concentration that would be provided via endotracheal tube, results in similar delivered concentrations to the lung that would be achieved with invasive delivery. Accordingly, while we cannot be sure that the effects of iNO delivered non-invasively were identical to the effects from invasively delivered iNO, it is likely that iNO concentrations delivered to the pulmonary vasculature were very similar in the two methods and had similar effects.

In the present study, long-iNO-treated infants weighing <750 g at birth (in whom long iNO did not increase BPD) received iNO for a mean duration of 52 ± 13 days, 9 days longer than the mean duration of long iNO-treated infants weighing 750–999 g at birth. Accordingly, it is unlikely that the duration of INO treatment is the cause of increased BPD in the subgroup of infants weighing 750–999 g at birth.

BPD is a heterogenous disease of prematurity, having components of lung parenchymal, large airway, and pulmonary vasculature disease of varying severities [23]. Multiple approaches to defining BPD and classifying its severity have been proposed, with the goals of determining associations with long-term outcomes, testing candidate treatments, and assessing changes in its epidemiology [18]. For this study, we defined BPD by oxygen- or positive pressure-dependence at 36 weeks PMA, and found that long iNO-treated babies had increased BPD, raising the concern that long iNO treatment had harmful, long-term effects. However, in a large, multi-center, retrospective study of the utility of different BPD definitions in predicting long-term outcomes in extremely preterm infants, the effectiveness of a BPD definition in predicting serious respiratory and neurosensory morbidities increased when the BPD definition used respiratory status at 40 weeks PMA compared to respiratory status at earlier post-menstrual ages [24]. Accordingly, it is reassuring that we found no difference between the groups in the number of infants requiring supplemental oxygen at 40 weeks PMA, either in the entire cohort or in the subgroup of infants weighing 750–999 g at birth—the subgroup that was entirely responsible for the finding in the entire cohort. This observation suggests that long iNO treatment was not ultimately harmful.

We observed clear differences in infants’ responses to long iNO depending on whether their mothers identified as Black or non-Black: in the entire cohort, long iNO treatment increased the incidence of BPD in non-Black infants, but did not increase BPD incidence in Black infants. Furthermore, long iNO treatment improved survival in infants weighing <750 g at birth, but only in infants identified as Black. Similar racial-identity-associated differences in responses of preterm infants to iNO treatment have been previously reported. In the NO-CLD study, in which 41% of infants were identified as Black or Hispanic, there was a significant interaction between racial identity and iNO treatment; the relative benefit of iNO in increasing survival without BPD was observed in Black and Hispanic infants but not in white infants [8]. More recently, an individual participant data meta-analysis of the three large, randomized placebo-controlled trials of iNO that used a starting iNO dose of greater than 5 ppm [7,8,9] reported a significant reduction in the composite outcome of death or BPD in Black infants, but not white or Hispanic infants [10]. The present study provides additional evidence that in the populations studied, iNO appears to differentially benefit preterm infants as a function of maternal racial identity.

How are these maternal racial identity-dependent differences in iNO benefits to be interpreted? Because racial identity is a social construct, racial identity-driven differences in treatment efficacy must be attributed to the social effects of racial identity, termed structural, or systemic, racism. As noted by Mulligan [25], “The scale and persistence of present-day racial inequities suggest that systemic racism and its effects have left their mark at multiple levels, impacting not just the community, family, and individual, but possibly also the epigenome”. Indeed, racial identity has been associated with the altered epigenetic modulation of fetal genes; the methylation of three CpG sites on two genes associated with preterm birth has been found to be lower in fetal DNA from Black women compared with non-Black women which may result in increased susceptibility to preterm birth [26]. Moreover, a recent study of nitric oxide-related gene expression identified inflammation and reactive oxygen species genes that are differentially expressed in Black women compared to non-Black women, supporting a racial identity-dependent association between genes in the NO pathway and inflammation-related microRNAs [27]. Our findings, and those of others, of differences in preterm infants’ responses to iNO as a function of maternal racial identity suggest that structural racism may have epigenetic effects on genes associated with NO production, such that supplemental iNO has a beneficial effect, either on survival without BPD, as others have reported, or on survival itself, as we report here. At the very least, our results support further study of the potential benefit of iNO treatment in Black populations in the US.

This study may be limited in its generalizability by having been performed at a single US center. Our study population, which was made up of a high proportion of infants born to Black, predominately socially disadvantaged mothers, provided a unique opportunity to identify the role of racial identity and structural racism in determining responses to iNO treatment. Nonetheless, our findings may have limited applicability to centers with more homogenous, non-Black populations or in societies without the structural racism endemic to the US.

## 5. Conclusions

In this randomized, double-blind, controlled trial, iNO treatment of very low birthweight, preterm infants requiring respiratory support from admission until 33 weeks gestation unexpectedly increased the combined incidence of death or BPD at 36 weeks PMA compared with iNO treatment for the first seven days of hospitalization. This increase was due entirely to increased BPD in the subgroup of infants weighing 750–999 g at birth. However, by the time this group of infants reached 40 weeks PMA, there was no difference in the use of supplemental oxygen between long and short iNO groups, a post-menstrual age when the effectiveness of a BPD definition in predicting serious respiratory and neurosensory morbidities is highest.

Long iNO decreased mortality in infants weighing <750 g at birth. This mortality decrease was seen only in Black infants. These findings suggest that iNO may specifically benefit Black preterm infants.

## Figures and Tables

**Table 1:** Characteristics of the intent-to-treat population.

	Short iNO (N = 138)	Long iNO (N = 135)	*p*
Birthweight, mean (SD)	921.1 (267.5)	913.0 (265.4)	0.80
Gestational Age, mean (SD)	27.2 (2.2)	26.8 (2.1)	0.11
Male (%)	75 (54.3)	72 (53.3)	0.90
Black (%)	99 (65.9)	91 (67.4)	0.51
Chorioamnionitis	15 (10.9)	21 (15.5)	0.29
Birthweight Group (%)			
<750 g	41 (29.7)	41 (30.4)	0.99
750–999 g	43 (31.2)	44 (32.6)	0.89
1000–1249 g	34 (24.6)	33 (24.4)	0.99
>1250 g	20 (14.5)	17 (12.6)	0.72
Birthweight < 10% percentile	12 (8.7)	10 (7.4)	0.70
Inborn (%)	119 (86.2)	119 (88.1)	0.72
Cesarean Section (%)	95 (68.8)	87 (64.4)	0.44
Apgar Score 1 min	5.0 (2.3)	4.7 (2.2)	0.27
Apgar Score 5 min	6.8 (1.8)	7.0 (1.6)	0.51
Antenatal Steroids (%)	116 (84.1)	109 (81.3)	0.54
Respiratory Illness Severity			0.99
Mechanical Ventilation	84 (60.9)	83 (61.5)	
CPAP	31 (22.5)	30 (22.2)	
Nasal Cannula	23 (16.7)	22 (16.3)	
Postnatal Infection	39 (28.3)	46 (34.1)	0.36

**Table 2:** The primary outcome and respiratory support-related secondary outcomes by treatment group.

	Short iNO (N = 138)	Long iNO (N = 135)	*p*	Relative Risk (95% CI)
BPD or Death (%)	65 (47.1)	83 (61.5)	0.017	1.37 (1.06–1.79)
Death	19 (13.8)	13 (9.6)	0.288	0.95 (0.87–1.04)
BPD	51 (37.0)	71 (52.6)	0.009	1.33 (1.07–1.66)
Supplemental O_2_ at 40 weeks PMA ^1^	30 (22.4)	28 (22.2)	0.97	1.00 (0.87–1.14)

^1^ Short iNO N = 134, long iNO N = 126.

**Table 3:** Analysis of the primary outcome of BPD or death by treatment and birth weight group.

Birthweight	Short iNO	Long iNO	*p*	Relative Risk (95% CI)
<750 g	36 (87.8) (N = 41)	37 (90.2) (N = 41)	0.72	1.25 (0.39–4.06)
750–999 g	20 (46.5) (N = 43)	36 (81.8) (N = 44)	<0.001	2.94 (1.54–5.90)
1000–1249 g	7 (20.6) (N = 34)	7 (21.2) (N = 33)	0.95	1.01 (0.77–1.32)
>1249 g	2 (10.0) (N = 20)	3 (17.6) (N = 17)	0.50	1.09 (0.82–1.55)

**Table 4:** Respiratory support-related secondary outcomes in infants weighing 750–999 g at birth.

	Short iNO (N = 43)	Long iNO (N = 44)	*p*	Relative Risk (95% CI)
BPD or Death (%)	20 (46.5)	36 (81.8)	<0.001	2.94 (1.54–5.90)
Death	4 (9.3)	6 (13.6)	0.52	1.05 (0.89–1.26)
BPD	17 (39.5)	30 (68.2)	0.007	1.90 (1.18–3.17)
Supplemental O_2_ at 40 weeks PMA ^1^	9 (21.4)	13 (34.2)	0.20	1.19 (0.91–1.62)

^1^ Short iNO N = 42, long iNO N = 38.

**Table 5:** Respiratory support-related secondary outcomes in infants weighing <750 g at birth.

	Short iNO (N = 41)	Long iNO (N = 41)	*p*	Relative Risk (95% CI)
BPD or Death (%)	36 (87.8)	37 (90.2)	0.72	1.25 (0.39–4.06)
Death	14 (34.1)	7 (17.1)	0.08	0.79 (0.59–1.03)
BPD	26 (63.4)	31 (75.6)	0.23	1.50 (0.78–2.95)
Supplemental O_2_ at 40 weeks PMA ^1^	17 (41.5)	15 (38.5)	0.78	0.95 (0.66–2.27)

^1^ Short iNO N = 41, long iNO N = 39.

**Table 6:** Incidence of the primary outcome of BPD or death and its components in Black and non-Black infants.

	Non-Black (N = 83)	Black (N = 190)	*p*	Relative Risk (95% CI)
BPD or Death (%)	45 (54.2)	103 (54.2)	0.99	0.99 (0.75–1.31)
Death	8 (9.6)	24 (12.6)	0.48	1.03 (0.93–1.12)
BPD	38 (45.8)	84 (44.2)	0.81	0.97 (0.76–1.21)
Supplemental O_2_ at 40 weeks PMA ^1^	13 (16.5)	45 (24.9)	0.13	1.11 (0.96–1.26)

^1^ Non-Black N = 79, Black N = 181.

**Table 7:** Incidence of the primary outcome and respiratory support-related secondary outcomes by treatment group in non-Black and Black infants.

Non-Black	Short iNO (N = 39)	Long iNO (N = 44)	*p*	Relative Risk (95% CI)
BPD or Death (%)	15 (38.5)	30 (68.2)	0.007	1.93 (1.20–3.24)
Death	4 (10.3)	4 (9.1)	0.86	0.98 (0.83–1.16)
BPD	12 (30.8)	26 (59.1)	0.01	1.69 (1.14–2.61)
Supplemental O_2_ at 40 weeks PMA ^1^	5 (13.2)	8 (19.5)	0.44	1.08 (0.87–1.34)
**Black**	**(N = 99)**	**(N = 91)**		
BPD or Death (%)	50 (50.5)	53 (58.2)	0.28	1.18 (0.87–1.63)
Death	15 (15.2)	9 (9.9)	0.28	0.94 (0.84–1.05)
BPD	39 (39.4)	45 (49.5)	0.16	1.20 (0.93–1.56)
Supplemental O_2_ at 40 weeks PMA ^2^	25 (26.0)	20 (23.5)	0.67	0.96 (0.81–1.15)

^1^ Short iNO N = 38; long iNO N = 41; ^2^ short iNO N = 96.

**Table 8:** Incidence of the primary outcome and respiratory support-related secondary outcomes by treatment group in non-Black and Black infants in infants weighing <750 g at birth.

Non-Black (<750 g)	Short iNO (N = 8)	Long iNO (N = 11)	*p*	Relative Risk (95% CI)
BPD or Death (%)	6 (75.0)	11 (100)	0.16	2.83 (0.86–5.78)
Death	2 (25.0)	2 (18.2)	0.72	0.92 (0.48–1.5)
BPD	5 (62.5)	9 (81.8)	0.35	2.06 (0.50–8.8)
Supplemental O_2_ at 40 weeks PMA ^1^	3 (37.5)	2 (20.0)	0.41	0.78 (0.37–1.44)
**Black (<750 g)**	**(N = 33)**	**(N = 30)**		
BPD or Death (%)	30 (90.9)	26 (86.7)	0.59	0.68 (0.18–2.54)
Death	12 (36.3)	5 (16.7)	0.08	0.76 (0.55–1.03)
BPD	21 (63.6)	22 (73.3)	0.41	1.57 (0.56–4.4)
Supplemental O_2_ at 40 weeks PMA ^2^	14 (42.4)	13 (44.8)	0.85	1.10 (0.41–2.91)

^1^ Long iNO N = 10; ^2^ long iNO N = 29.

**Table 9:** Incidences of secondary outcomes in the treatment groups.

	Short iNO (N = 138)	Long iNO (N = 135)	*p*
Severe IVH/PVL (%)	13 (9.4)	16 (11.9)	0.56
Pulmonary hemorrhage	2 (1.4)	5 (3.8)	0.28
Pneumothorax, n (%)	13 (9.4)	10 (7.4)	0.66
PIE (%)	19 (13.8)	26 (19.2)	0.26
Symptomatic PDA (%)	45 (32.6)	41 (30.4)	0.80
NEC, Bell’s stage ≥ 2 (%)	14 (10.1)	19 (14.1)	0.20

## Data Availability

The data presented in this study are not freely available.
